# Internal validation of an automated system for brachial and femoral flow mediated dilation

**DOI:** 10.1186/s40885-017-0073-1

**Published:** 2017-08-22

**Authors:** Brycen Ratcliffe, Robert Pawlak, Francisco Morales, Caleb Harrison, Alvaro N. Gurovich

**Affiliations:** Department of Applied Medicine and Rehabilitation, Indiana State University, Sycamore Center for Wellness & Applied Medicine, 567 North 5th Street, Terre Haute, IN 47809 USA

**Keywords:** Flow mediated dilation, Coefficient of variation, Validation, Endothelial function

## Abstract

**Background:**

Flow Mediated Dilation (FMD) has immense potential to become a clinical, non-invasive biomarker of endothelial function and nitric oxide bioavailability, which regulate vasomotor activity. Unfortunately, FMD analysis techniques could deviate significantly in different laboratories if a validation process is not involved. The purpose of this study was to provide validation to the assessment of FMD analysis in our laboratory and to standardize this process before reporting results of FMD.

**Methods:**

Brachial and femoral arteries FMD were performed on 28 apparently healthy participants (15 male and 13 female, ages 18–35 years). For the intratester reliability study, nine subjects were asked to come to the lab for a second brachial FMD within 48 h. All FMD procedures were performed by the same investigator, while the FMD analyses were performed by 2 independent testers who were blind to each other’s analyses. FMD analyses included baseline artery diameter measurements, peak artery diameter after 5 min of ischemia, and FMD. Analysis was completed via an automated edge detection system by both testers after training of the methodical process of analysis to minimize variability. Intratester and intertester reliability were determined by using coefficient of variation (CV) between first and second visit (intratester) and between results obtained by both testers (intertester).

**Results:**

The intratester CVs for tester 1 and 2 were 3.28 and 2.62%, 3.74 and 3.27%, and 4.95 and 2.38% for brachial baseline artery diameter, brachial peak artery dilation, and brachial FMD, respectively. In the intertester CVs were 2.40, 3.16, and 3.37% for brachial baseline artery diameter, peak artery dilation, and FMD, respectively and 4.52, 5.50, and 3.46% for femoral baseline artery diameter, peak artery dilation, and FMD, respectively.

**Conclusion:**

All CVs were under or around 5%, confirming a strong reliability of the method. Our laboratory has shown that the FMD protocol is reproducible due to the significantly low coefficient of variation. This is one step closer to use FMD as a biomarker for endothelial function in our laboratory.

## Background

Atherosclerosis, the pathological deterioration of endothelium that results in vessel blockage, is responsible for approximately 90% of all cardiovascular diseases [1, 2]. Endothelial dysfunction is the first step in this process, defined by the decrease in nitric oxide availability within the endothelium, developing oxidative stress. When endothelial oxidative stress is increased, endothelial NO synthase is uncoupled, and generates a decrease in NO production and bioavailabilty [3]. Flow Mediated Dilation (FMD) is a non-invasive assessment of endothelial function and vasodilatory capacity in humans [4–6] with an immense potential to become a clinical tool for cardiovascular risk assessment [1]. Therefore, FMD is normally considered as a biomarker for endothelial function and NO bioavailability in humans, relevant to study cardiovascular risk factors such as hypertension [5, 7]. There are three major sites where FMD is assessed. The most studied one is the brachial artery [4, 5]; however, femoral artery [8] and popliteal artery [9] sites have been used to study large elastic and leg conduit arteries, respectively.

In general, there is a consensus on how to perform FMD [5, 10, 11], however, there are several analysis systems that can be used to determine vessel diameters [12–17]. In addition, FMD analysis could significantly deviate in different laboratories which makes the comparison of results very difficult. To provide a better understanding of the analyzed data, each laboratory must standardize their specific protocols and provide information about technical error of the procedure.

Therefore the purpose of this study is to provide validation to the assessment of FMD analysis in our laboratory and to standardize this process as a first step in every laboratory prior to reporting results of FMD.

## Methods

Thirty apparently healthy individuals, 15 males and 15 females, ages 18–35 years old, were recruited for this study. Exclusion criteria included known cardiovascular or cardiac disease, prescription medication medicines, and ‘over-the-counter’ painkillers, such as NSAIDs or aspirin, or nutritional supplements containing antioxidants [7]. Young female participants were tested within 4 days before or within 4 days after menses to standardize any influence that certain phases of the menstrual cycle may have on vascular physiology [18].

The investigation was approved by the Indiana State University Institutional Review Board for ethical practices and declared in accordance with the Declaration of Helinski. All subjects were tested at the same time of day to avoid any diurnal variations and following at least 8 h of fasting [5, 11].

### Design

All participants completed a single laboratory testing session assessing baseline blood pressure (BP), heart rate (HR), and endothelium-dependent vasodilation via flow mediated dilation procedures. All laboratory testing was conducted in a darkened, quiet and temperature-controlled room approximately at 24 °C. All patients were required to abstain from food and caffeine for at least 8 h prior to testing, and a 12 h abstinence from exercise. Ten participants were asked to return for a second visit within 48 h after the initial assessment to repeat the procedure.

### Procedure

#### Brachial and femoral flow mediated dilation

Participants were asked to relax and lay down on an examination table. Following a 10-min rest period brachial blood pressure was measured in triplicate via an automated non-invasive device (Omron Automatic Blood Pressure Monitor, Omron Healthcare, Lake Forest IL). After assessing hemodynamic baseline conditions, a blood pressure cuff was placed on the participants’ upper forearm of their right arm, just below the antecubital region of the elbow, or on the upper lower leg of the right leg, just below the patella, for brachial (b) or femoral (f) FMD, respectively. After baseline measurements were obtained, the blood pressure cuff was then inflated to a supra systolic pressure (>200 mmHg) for 5 min, which was sufficient to provide ischemia and subsequent reactive hyperemia to the distal end of the arm and leg. FMD was performed using high-resolution ultrasound (GE Logiq E, GE Medical, Milwaukee, WI). A 12.0 MHz linear phase array ultrasound transducer was used to image the right brachial and femoral arteries longitudinally. According to recent guidelines [5], using a 12 MHz ultrasound probe during the imaging process allows for a better definition of the endothelium at the brachial and femoral artery; however, ultrasound frequencies over 10 MHz also provide good definition, especially when using edge-detection technology. Baseline brachial and femoral artery diameter measurements were obtained utilizing an electrocardiogram (ECG) trigger system (MP150WSW, BIOPAC Systems Inc., Goleta, CA and Frame Grabbing and Digital Data Input modules, Medical Imaging Applications LLC, Coralville IA). Using an ECG-gated image selection is more time efficient as the live video stream from the imaging ultrasound feeds directly on a digital recording device using one frame per cardiac cycle. Even though previous reports have selected one frame every 3 to 5 s [8] and even every 15 s [4], the use of ECG-gated image selection decreases the chances to miss the peak dilation, improving accuracy.

Imaging was performed with the ultrasound probe fixed approximately 5 cm above the antecubital fossa and approximately 2 cm below the inguinal ligament for brachial and femoral measurement sites, respectively. All images were recorded continuously for 180 from 30 s prior to cuff deflation and stored as AVI files for off-line analysis, which was performed using an automated edge-detection software (Vascular Research Tools, Medical Imaging Applications LLC, Coralville IA). Brachial and femoral peak diameters were identified as the single peak diameter observed during the plateau phase after cuff deflation. All FMD procedures were completed by the same investigator (ANG) in accordance with published guidelines [5, 11].

### Analysis

**Fig. 1:**
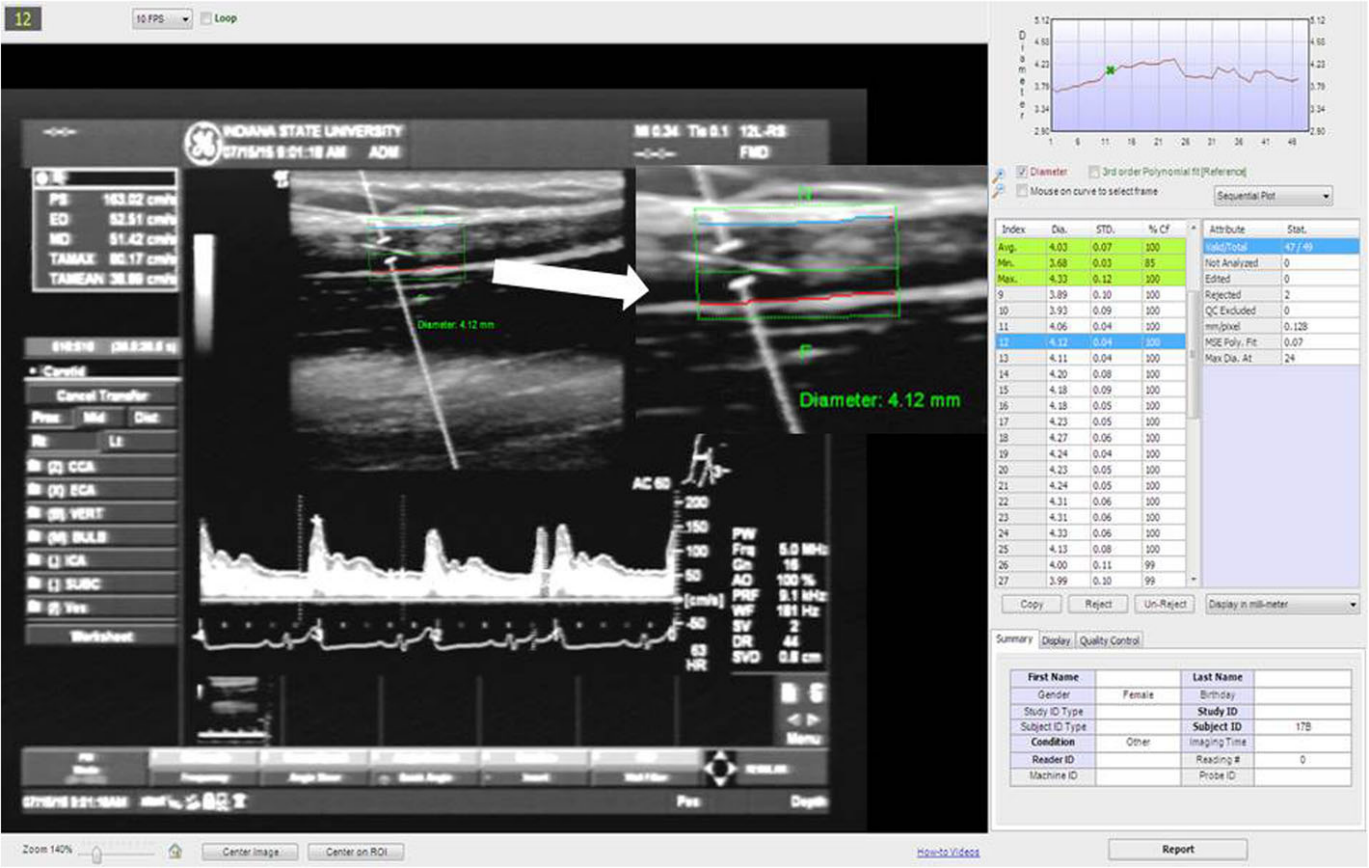
Screenshot of a representative brachial FMD analysis using edge-detection technology. On the left-hand panel is the analyzed frame from the ultrasound imaging system and on the right-hand panel are the actual measurements from frame 9 to 27, including a time-based graph on the overall FMD on the right-top-panel. On the middle, the region of interest (ROI) has been zoomed for better view

Analysis was performed by two independent testers (BR and RP) who were blind to each other’s analyses. Analysis included baseline artery diameter measurement, peak artery diameter post-ischemia, as well as FMD. Analysis was completed via Brachial Analyzer for Research® Software (Medical Imaging Applications LLC, Coralville, Iowa) by both testers after training of the methodical process of analysis to minimize variability (Fig. 1). After reviewing FMD images, a square Region of Interest (ROI) was placed on the most stable region of the artery. The ROI encompassed both sides of the endothelial lumen and measured a width of at least 3 mm ± 0.1 mm across. The ROI was then locked, and curvature of ROI to endothelial lumen was checked for consistent diameter accuracy. Images with less than 75% Confidence Index of quality control were edited with computerized assistance to best fit arterial morphology. No more than nine computerized assistances were allowed, if more were needed for accurate results, the frame was rejected. Standard deviation of the measured frame was minimized when possible, maximal value allowed was 0.15 mm.

In this experiment, FMD calculations were allometrically scaled using FMD = [Peak Diameter/(Baseline Diameter^0.89^)] proposed by Atkinson et al. [19, 20] after analyzing the slope of the relationship between logarithmic transformed baseline and peak diameters for this specific sample. In addition, FMD is presented as a percentage change from baseline measurements (%FMD) using the following formula:

$$ \%FMD=\left[\left(\mathrm{Peak}\ \mathrm{Diameter}\hbox{--} \mathrm{Baseline}\ \mathrm{Diameter}\right)/\left(\mathrm{Baseline}\ \mathrm{Diameter}\right)\right]\ \mathrm{x}\ 100. $$

### Statistical analysis

All variables were checked for normal distribution using Kolmogorov-Smirnov and descriptive statics, including mean and standard deviations, were obtained. A two-way ANOVA (Tester x Sex) was performed to determine differences in both brachial and femoral %FMD between testers and males and females. Intratester analysis was performed using collected data from subjects attending both brachial FMD sessions. Intertester analysis was performed using data from all 30 subjects comparing analyses between testers in both brachial and femoral assessments. Validation of the analysis technique was determined by using both intratester and intertester coefficient of variation (CV). In addition, t-tests were performed to compare intertester diameters and FMD as well as intratester CVs. All statistical analyses were performed using SPSS version 23.0 (IBM, Chicago, IL), and statistical significance was considered when *p* < 0.05.

## Results

During the study, data collected during brachial FMD from one subject had some technical error on the first visit. In addition, data collected during femoral FMD from two subjects had some technical error. Data from these subjects were withdrawn from the intertester and intratester analyses. All data was normally distributed according to Kolmogorov-Smirnov analysis.

Table 1 shows the general characteristics of the sample. Males were taller, heavier, had a larger BMI, and higher systolic blood pressure than females (*p* < 0.05).

**Table 1:** Sample demographics

	Total (*n* = 28)	Males (*n* = 15)	Females (*n* = 13)	*P* value
Age (yrs)	24.01 ± 4.28	24.14 ± 4.88	23.87 ± 3.75	0.868
Height (m)	1.71 ± 0.09	1.77 ± 0.06	1.65 ± 0.08	<0.001^*^
Weight (kg)	73.36 ± 17.51	84.63 ± 17.2	62.1 ± 8.19	<0.001^*^
BMI (kg/m^2^)	25.09 ± 4.97	27.11 ± 5.36	23.08 ± 3.70	0.023^*^
Systolic Blood Pressure (mmHg)	109.53 ± 10.48	115.00 ± 9.29	104.07 ± 8.80	0.003^*^
Diastolic Blood Pressure (mmHg)	70.37 ± 8.53	70.00 ± 7.62	70.73 ± 9.62	0.819

^*^statistically significant between males and females

There were no significant differences between males and females and between testers on both brachial and femoral %FMD (Table 2). In general, brachial %FMD is larger than femoral %FMD, which could be attributed to a smaller baseline diameter observed in the brachial artery.

**Table 2:** Brachial and femoral percentage of flow mediated dilation in males and females analyzed by two independent testers

	Total	Males	Females	Tester Effect	Sex Effect	Interaction
Brachial FMD (%) Tester 1	14.9 ± 8.5	16.3 ± 8.8	33.5 ± 8.3	0.07	0.26	0.72
Brachial FMD (%) Tester 2	11.5 ± 5.2	12.2 ± 4.3	10.8 ± 6.0			
Femoral FMD (%) Tester 1	8.2 ± 8.2	6.9 ± 5.8	9.4 ± 10.0	0.48	0.34	0.83
Femoral FMD (%) Tester 2	9.7 ± 7.5	8.9 ± 4.2	10.5 ± 9.8			

Intratester analysis is shown on Table 3. CVs were all below 5.0% and there were no significant differences between CVs between tester 1 and tester 2 in any of the studied variables.

**Table 3:** Intratester analysis on both testers (*n* = 9)

	Tester 1 (%CV)	Tester 2 (%CV)	P
Brachial baseline	3.28 ± 3.63	2.62 ± 2.61	0.68
Brachial peak	3.74 ± 4.90	3.27 ± 2.01	0.75
Brachial FMD	4.95 ± 5.83	2.38 ± 1.79	0.26

%CV: coefficient of variation in percentage

Intertester analysis is shown on Table 4. CVs were all at or below 5.5% and there was only one significant difference between both testers (brachial FMD).

**Table 4:** Intertester analysis (*n* = 28)

	Tester 1	Tester 2	P	(%CV between T1 and T2)
Brachial baseline (mm)	3.62 ± 0.56	3.60 ± 0.59	0.89	2.40%
Brachial peak (mm)	4.14 ± 0.51	4.00 ± 0.59	0.37	3.16%
Brachial FMD (ratio)	1.32 ± 0.86	1.28 ± 0.55^*^	0.04	3.25%
Femoral baseline (mm)	5.95 ± 1.16	6.20 ± 0.91	0.39	4.52%
Femoral peak (mm)	6.41 ± 1.16	6.78 ± 0.89	0.20	5.50%
Femoral FMD (ratio)	1.31 ± 0.95	1.34 ± 0.83	0.30	3.61%

^*^statistically significant difference between tester 1 and tester 2

## Discussion

The present study was designed to internally validate the analysis of FMD within the same tester and between testers. The major findings in this study are two-fold: first, the FMD analysis protocol is reproducible within same tester and second, the FMD analysis protocol is reproducible between testers.

In general, FMD protocols follow international guidelines [5, 10, 11]. Our laboratory is not an exception. All images are obtained with an isonation angle of 60° and images are obtained with the help of an ECG-gated system to avoid changes of vessel diameter observed within one cardiac cycle. These technical details provide more accurate and clear images of the studied vessel. The recording duration of 180 s, 30 s prior to deflation accompanied by 150 s post-deflation, was determined to be the most adequate method in which FMD could be determined [5, 10]. The 30 s prior to deflation allows to record images of the vessel that reflect differences with baseline diameters [7, 21]. After the cuff deflation, the hyperemic blood flow will rapidly increase eliciting the vascular response, which will peak anywhere between 30 and 90 s in an average 18–35 years old adult. The additional 60 s help to account for outliers in the vasodilatory response as well as return to baseline measurements.

The present study showed that the intratester CVs from two independent testers (tester 1 and 2) were very similar in all studied variables (range 2.62–4.95%, Table 3) and there no significant difference between the CVs from both testers. Brachial baseline and peak CVs are all below 3.75%, while FMD CV was slightly higher in one of the testers (4.95%). The CVs obtained in the present study are lower than the CVs from previous reports, which are already considered as low methodological error [16, 17, 22]. Therefore, the intratester validation presented here provides data for brachial baseline and peak artery dilation, making this data an acceptable reproducible measurement of brachial artery recording. Similar results were observed for the intertester analysis (Table 4). CVs between both testers were lower or at 5.50% for all studied variables. All the studied diameters, brachial baseline and peak and femoral baseline and peak, and femoral FMD showed no significant difference between testers. Only brachial FMD showed a significant difference between testers. This difference might be the results of slightly larger peak brachial diameter observed on tester 1, which could produce a larger difference when the FMD ratio is applied. Interestingly, and according to our best knowledge, this is the first study designed to determine reproducibility between two independent testers. The results of the present study show that the FMD protocol use in our laboratory is reproducible within a ~ 5% of variation between testers.

Other studies using similar approaches have shown comparable results than the present study. For example, Avery et al. [16] used the same automated edge detection system [15] as the present study. Their CVs were slightly higher than 5% (4–10%). This difference might be explained by the difference on their acquisition system, a 10.5 MHz ultrasound probe, which could decrease the image definition [5, 15]. Woodman et al. [17], using a tailored automated edge detection system, also found slightly higher CVs than the ones presented here (6.7%). Interestingly, their image acquisition was also performed with a 10 MHz ultrasound probe, which can produce less defined images [5]. Finally, Ghiadoni et al. [12, 22] used a new automated edge detection system that does not use ECG-gated image selection, using the average vessel diameter within a cardiac cycle. In addition, they recommend using 7.5 to 10 MHz ultrasound probes, which decreases images definition [5]. These two major differences might be responsible of higher CVs (7.6–11.6%) than the ones observed in the present study [22].

The final impact that this study had was the reproducibility of the observed results. A major factor of clinical utilization of FMD as a diagnostic technique is the accurate replicability between analyzers [1, 5, 11]. In this laboratory, two individuals with different backgrounds in research performed this validation study. One tester was a research assistant for nearly 2 years in this lab, while the other had been working in the lab for only 1 month prior to this analysis. The same techniques were provided by the principal investigator to both analyzers, with the same allotted practice time and techniques given from previous studies [17, 23]. The correlation between testers provided by this data proves the reproducibility of this laboratory procedure and advances the application of FMD as a future clinical diagnostic tool.

In essence, the present study was designed to provide a basis of support and assess the capabilities of our laboratory to provide accurate analysis and a specific, reproducible protocol for FMD testing. One of the main problems with FMD becoming a clinical assessment tool is the wide variety of results between laboratories and the dependency upon experimenter knowledge and background [23]. To solve the problem, our laboratory has created a structured methodological approach, accompanied by several sessions of background information about FMD that discussed background knowledge, methods, and common misconceptions, including image acquisition, recordings storage, and analysis software training. The present approach provides a basis of support for the protocol being easily reproducible and helps laboratories performing FMD to use it as a potential biomarker.

This study was not without limitations. First, the present study used the calculation of FMD proposed by Atkinson et al. [19, 20, 24]. This method deviated from previous studies, which used %FMD = [(Peak Diameter – Baseline Diameter)/(Baseline Diameter)] × 100 and implements a mathematical correction to account for the confounding influence of allometric scaling between individuals [19, 20, 23, 24]. Atkinson et al. approach allows constructing a mathematically efficient and more accurate representation of FMD ratio, which in the present study are different from data presented from other laboratories [4–6, 11, 16, 17, 22]. Secondly, the ECG-gated trigger system produced some incorrect readings on the QRS complexes, sometimes choosing a frame picture that was not at the correct time line. However, this limitation was non-significant as an occurrence in less than 1 in 1000 frames was observed. Finally, one individual, as highlighted in the results section, had to be removed from the intratester analysis due to technical difficulties that could not be amended, limiting our number of participants (*n* = 9).

## Conclusion

The present study shows that in this laboratory, the described protocol is reliable and reproducible. All Coefficients of Variation (CV) were under or approximately 5%, confirming a strong reliability of the protocol and methods associated. CV was very similar between Tester 1 and Tester 2 and there was no statistical significance between Tester 1 and Tester 2 and between both testers’ CVs. The present study should promote the concept of all laboratories validating FMD procedures before carrying out any further FMD testing in order to obtain a reproducible and validated result in any future procedure that utilizes FMD. Finally, our laboratory will use the present study as a current reference of our FMD protocol reliability in any further scientific communication.

## Abbreviations

BMI: Body mass index
BP: Blood pressure
CV: Coefficient of variation
ECG: Electrocardiogram
FMD: Flow mediated dilation
HR: Heart rate
NO: Nitric oxide
ROI: Region of interest
